# Exploring the diagnostic potential of IL1R1 in depression and its association with lipid metabolism

**DOI:** 10.3389/fphar.2025.1519287

**Published:** 2025-04-24

**Authors:** Yao Gao, Xiao-Na Song, Nan Zhang, Huang-Hui Liu, Jian-Zhen Hu, Xin-Zhe Du, Guo-Hua Song, Sha Liu

**Affiliations:** ^1^ Department of Psychiatry, First Clinical Medical College/First Hospital of Shanxi Medical University, Taiyuan, China; ^2^ Shanxi Key Laboratory of Artificial Intelligence Assisted Diagnosis and Treatment for Mental Disorder, First Hospital of Shanxi Medical University, Taiyuan, China; ^3^ Department of Basic Medical Sciences, Shanxi Medical University, Taiyuan, China; ^4^ Laboratory Animal Center, Shanxi Medical University, Taiyuan, China; ^5^ School of Pharmaceutical Science, Shanxi Medical University, Taiyuan, China

**Keywords:** depression, Il1r1, oxidative stress, lipid metabolism, diagnostic potential, mendelian randomization, drug target

## Abstract

**Background:**

Depression is a complex mental disorder where oxidative stress and lipid metabolism disorders play crucial roles, yet their connection requires further exploration. This study aims to investigate the roles of oxidative stress and lipid metabolism disorders in depression using bioinformatics methods and Mendelian randomization analysis.

**Methods:**

A differential gene expression analysis was performed on the GSE76826 dataset, followed by identification of the intersection with genes related to OS. Subsequently, support vector machine (SVM) and random forest algorithms were employed to determine the optimal division of feature variables. The diagnostic performance was evaluated using a ROC diagnostic model and Diagnostic Nomogram. Furthermore, Mendelian randomization (MR) analysis was conducted to explore the causal relationship between the gene and depression. The expression patterns of key genes in brain tissue were analyzed using the Human eFP Browser database, while their associations with metabolism-related genes were investigated using the STRING database. Finally, DrugnomeAI was utilized to assess the drug development potential of these genes, and small molecule compounds targeting them were identified through dgidb and ChEMBL databases; molecular docking studies were then conducted to evaluate their binding affinity.

**Results:**

By conducting a comprehensive analysis of oxidative stress-related genes and depression-related target genes, we have successfully identified 12 overlapping genes. These 12 genes were selected using support vector machine and random forest algorithms. Upon analyzing the diagnostic model, it was revealed that EPAS1 and IL1R1 serve as key biomarkers for OS in depression, with IL1R1 exhibiting the highest diagnostic potential among them. Additionally, MRfen analysis suggests that IL1R1 may play a protective role against depression. Notably, this gene exhibits high expression levels in crucial brain regions such as the olfactory bulb, corpus callosum, and hippocampus. Furthermore, our findings indicate an association between IL1R1 and lipid-related genes PDGFB, PIK3R1, TNFRSFIAA NOD2, and LYN. DrugnomeAI analysis indicated promising medicinal value for ILIRI with BI 639667 demonstrating superior binding affinity among the selected small molecule drugs.

**Conclusion:**

This study provides novel insights into the association between OS and dyslipidemia metabolism in depression, offering potential therapeutic targets for future drug development.

## 1 Introduction

Depression, a prevalent mental health disorder, is characterized by persistent sadness, diminished interest in daily activities or anhedonia, and is accompanied by a range of emotional and physical symptoms. These symptoms significantly impact the patient’s overall quality of life (Liu et al., 2023). According to epidemiological survey data, the lifetime prevalence rate of adult depression disorders in China is 6.8%, with depression accounting for 3.4%. The number of individuals suffering from depression in China amounts to 95 million, while approximately 280,000 people commit suicide annually; among them, 40% are affected by depression. The etiological mechanism underlying depression is highly intricate and involves the interplay between various complex factors such as neurobiology, genetics, and social psychology. Although several theories have been explored regarding the causes of major depression—such as the monoamine hypothesis, OS hypothesis, neurotransmitter hypothesis, neuroinflammatory hypothesis, and neuroplasticity hypothesis—none has comprehensively encompassed the multifaceted nature of this disorder (Cui et al., 2024).

The OS hypothesis of depression posits that elevated levels of reactive oxygen species (ROS) and diminished antioxidant defenses alter brain structure (Chai et al., 2023), which is closely associated with the onset of depression (Lu et al., 2022; Ait Tayeb et al., 2023). Under normal physiological conditions, a delicate equilibrium is maintained between the oxidant and antioxidant systems. However, when ROS production surpasses the clearance capacity of the antioxidant reaction system, it results in substantial protein oxidation and lipid peroxidation. Excessive accumulation of ROS can lead to oxidative damage, cellular degeneration, and decline in physiological function (He et al., 2017). Furthermore, as OS intensifies, activation of pro-inflammatory signaling pathways also plays a pivotal role in the pathogenesis of depression (Dang et al., 2022). Major depressive disorder (MDD) is characterized by an imbalance between neurodegenerative and neuroprotective factors such as brain-derived neurotrophic factor (BDNF) and nuclear factor (NF)-κB (Fries et al., 2023). Moreover, MDD is associated with elevated levels of pro-inflammatory cytokines in various inflammatory processes, diminished nerve growth, and subsequent neuronal degeneration. The production of interleukin-1 (IL-1) and interleukin-18 (IL-18), key inflammatory markers, along with the formation of cell membrane pores and intracellular substance leakage can all contribute to cellular apoptosis (Syed et al., 2018). Recent research has emphasized the crucial role of inflammation in the pathophysiology of major depression and established a connection between pro-inflammatory cytokines and depressive symptoms. For instance, these cytokines have been demonstrated to disrupt monoaminergic neurotransmission and synaptic plasticity, both vital for mood regulation (Kobayashi et al., 2022; Brock et al., 2023). Neuroimaging studies further support the notion that neuroinflammatory processes impact brain connectivity and function in individuals with major depression by revealing alterations in key mood-regulating brain regions such as the hippocampus and prefrontal cortex (Chen et al., 2021). Additionally, oxidative stress-induced free radicals and reactive oxygen species can attack lipid components on cell membranes leading to lipid peroxidation which subsequently impairs cellular structure and function (Tauffenberger and Magistretti, 2021). Such disruption not only affects cell membrane permeability but also interferes with intracellular signal transduction pathways and metabolic processes. Therefore, OS plays a significant role in MDD (Patel, 2016; Cobley et al., 2018). Despite advancements in research, inconsistent findings coupled with the multifactorial nature of major depression have resulted in substantial gaps remaining regarding our understanding of how these biological mechanisms interact to manifest clinical symptoms.

Moreover, the heterogeneity of MDD presents significant diagnostic and therapeutic challenges. The prevalence of treatment-resistant patients underscores the pressing need for innovative therapeutic strategies to overcome the limitations associated with conventional monoamine interventions (Sun et al., 2022). Emerging approaches, including neuromodulation techniques and psychedelic utilization, have demonstrated promising preliminary outcomes in clinical trials; however, further investigation is required to elucidate their mechanisms of action and long-term efficacy in greater detail (Marwaha et al., 2023). Given the inherent complexity of MDD, a multidimensional interdisciplinary approach that integrates insights from diverse fields such as genomics, epigenetics, and neurobiology is essential for achieving a comprehensive understanding of its underlying mechanisms (Fries et al., 2023).

In this study, we employed bioinformatics techniques to identify and validate key genes associated with depression. By conducting comparative analysis of differentially expressed genes (DEGs) in samples from healthy individuals and depressed patients, coupled with Kyoto Encyclopedia of Genes and Genomes (KEGG) enrichment analysis, we investigated the biological functions and pathways implicated in these genes. Furthermore, we performed cross-analysis between depression-related genes and oxidative stress-related genes to assess their diagnostic potential. Additionally, MR was utilized to analyze the association between core genes and depression. Finally, leveraging multiple databases and bioinformatics tools, we explored the druggability of these key genes to preliminarily identify potential therapeutic targets for future drug development endeavors. Overall, this study presents a comprehensive analysis of pivotal genes and molecular pathways involved in depression, offering novel insights into disease mechanisms as well as potential therapeutic targets. By integrating genomics data with bioinformatics approaches, our aim is to bridge the gap between gene discovery and clinical application in order to enhance diagnostic accuracy and treatment outcomes for depression.

## 2 Materials and methods

### 2.1 Data sources

The GEOquery package was utilized to download the dataset from the GSE76826 GEO database (https://www.ncbi.nlm.nih.gov/) (Miyata et al., 2016). This dataset examined biomarkers in older outpatients and inpatients with Major Depressive Disorder (MDD), specifically those aged 50 years and above. The depressive status of participants was assessed using the Hamilton Depression Structured Interview Guide (SIGH-D) rating scale. Subsequently, the normalizeBetweenArrays function from the limma package was employed for further normalization of the data. Following this, differential expression analysis of genes between 12 normal samples and 10 patients diagnosed with depression was conducted using the limma package. Differentially expressed genes were identified based on criteria of |log2FC| > 1.2 and *P* < 0.05. The results of this differential analysis were visualized through a volcano plot. During annotation processing, probes corresponding to multiple molecules were removed, ensuring that only unique probes remained for each molecule encountered; among these, only the probe exhibiting the highest signal value was retained. Finally, KEGG enrichment analysis was performed on the selected differentially expressed genes. Additionally, GWAS data on depression (dataset finn-b-F5_DEPRESSIO) comprising23,424 European individuals and covering 8,281,749 Sips was obtainedfrom the IEU Open GWAS database (https://gwas.mrcieu.ac.uk/datasets/finn-b-F5_DEPRESSIO/).

### 2.2 Screening of potential targets

The genes associated with OS were screened in the GSEA database. Subsequently, an intersection analysis was conducted between depression-related target genes and oxidative stress-related target genes. To optimize the classification of these intersecting genes, two machine learning algorithms, namely, support vector machine (SVM) and random forest, were employed for comprehensive analysis and processing. The R software [4.2.1] was utilized for data analysis, employing the random Forest package (version 4.7.1.1) to construct a random forest model aimed at evaluating feature importance. Initially, a random seed (seed = 2024) was established to ensure the reproducibility of results. Following the framework of the random forest algorithm proposed by Breiman (Breiman, 2001), an ensemble model comprising 100 decision trees was developed using default parameter settings for data analysis. The Mean Decrease Accuracy of each feature was computed to quantify its importance score during model training. Simultaneously, SVM (Meyer et al., 2015) analysis was conducted utilizing the e1071 package [1.7.13] for classification modeling purposes. A random seed (seed = 2024) was preset prior to data processing, ensuring repeatability in the calculation process through the set.seed() function. When constructing the classification model with the svm() function, a 5-fold cross-validation strategy was employed to assess model stability effectively. The Gaussian radial basis kernel was selected as the algorithm’s kernel, facilitating nonlinear mapping to handle complex data structures efficiently. Results were visualized using the ggplot2 package [3.4.4].

### 2.3 Biomarker screening for depression

To assess the diagnostic value of a gene, we employ receiver operating characteristic (ROC) analysis to construct a diagnostic model and subsequently validate its performance using a Diagnostic Nomogram. The gene-based nomogram was constructed using the “rms' package. Subsequently, a binary logistic model was built using the glm function, and model calibration analysis and visualization were performed using the rms [6.4.0] package. The rmda package was utilized to calculate the corresponding net return rate and facilitate visual display, with results visualized through ggplot2 [3.3.6].

### 2.4 Tool variable filtering

In the two-way MR analysis, a screening threshold of P < 1 × 10^−5^ was applied to ensure an adequate number of SNPs. Subsequently, instrumental variables (IVs) exhibiting strong linkage disequilibrium (LD) were excluded based on r2 < 0.001 and kb = 10,000 criteria. The F statistic was then calculated as F = [R2/(1-R2)] × (N-K-1)/K, where R2 represents the proportion of exposure factor change explained by IVs, N denotes the sample size associated with the exposure factor, and K represents the number of instrumental variables used. If F < 10 is considered indicative of weak instrumentality, it should be eliminated.

### 2.5 Two-sample MR analysis method

In order to investigate the causal relationship between depression and key genes, this study employed a two-sample MR analysis. The instrumental variable single nucleotide polymorphism (SNP) used in this analysis should satisfy the assumptions of “correlation with exposure factors', which include being correlated with exposure factors, unaffected by confounding factors, and only affecting outcome factors through exposure factors. Key genes related to the phenotype were screened from the National Center for Biotechnology’s database (https://www.ncbi.nlm.nih.gov/). A GWAS aggregate statistical dataset available on the MR Platform was utilized, consisting of 23,424 individuals of European descent as phenotypes for depression. Various methods including MR Egger, weighted median, inverse variance weighting (IVW), simple model, and weighted model were employed to conduct two-sample MR analyses. The IVW results served as the primary evaluation indicators while other method results estimating causal effects were considered sensitivity analyses. Statistical significance was determined if *P* < 0.05. To facilitate result interpretation, Beta values obtained in this study were converted into odds ratios (OR), accompanied by calculation of their corresponding 95% confidence intervals (CI).

If the instrumental variable affects the outcome through factors other than the exposure factor, it indicates the presence of horizontal pleiotropy, which undermines the validity of the assumptions of independence and exclusivity. To assess horizontal pleiotropy, we employ MR-Egger intercept analysis. A P-value greater than 0.05 suggests no statistically significant deviation from zero intercept, indicating that the results are not influenced by horizontal pleiotropy and confirming that the instrumental variable solely impacts the outcome through exposure.

### 2.6 Expression patterns of pharmaceutically usable genes in brain tissue

Numerous studies have demonstrated that depression is a psychiatric disorder characterized by aberrant functionality in specific cerebral regions. In this investigation, the therapeutic potential of medicinal gene intervention for depression was explored using the Human eFP (“electronic fluorescent hieroglyphs') browser (http://bar.utoronto.ca/efp_human/), which facilitated rapid examination of gene expression profiles and intuitively revealed the diverse brain regions expressing IL1R1.

### 2.7 Relationship between key genes and lipid-related genes

The GSEA database was utilized to screen for genes associated with lipid metabolism, enabling the analysis of differential gene expression related to lipid metabolism in depression. Additionally, the STRING data facilitated network interaction analysis of these differentially expressed genes. GSE76826 data set was used for Spearman correlation analysis of differential genes.

### 2.8 Pharmaceutically available gene-related drug screening and molecular docking and modeling

We employed DrugnomeAI (https://astrazeneca-cgr-publications.github.io/DrugnomeAI/) to assess the medicinal potential of IL1R1, considering a target with a percentile score >60 in DrugnomeAI gene rankings as having promising medicinal properties. Subsequently, we utilized the dgidb follow-up database (https://www.dgidb.org/), ChEMBL database (https://www.ebi.ac.uk/chembl/), and relevant literature searches on gene-drug interactions to evaluate the effects of potential drug candidates on IL1R1. Furthermore, stringent criteria for safety, efficacy, and ethical approval were applied to select suitable drug candidates.

We utilized AutoDockTools-1.5.7 software to conduct molecular docking of the medicinal gene with the potential drug candidate and determine the binding energy, which served as an indicator for evaluating the binding activity between pharmaceutically available genes and drug candidates. A higher absolute value of binding energy indicates better binding activity and stability. Specifically, a binding energy ≤ −5.0 kcal/mol suggests a more favorable interaction between pharmaceutically available genes and drug candidates. The results demonstrated that the combination of the medicinal gene and candidate drug exhibited significant efficacy.

Molecular dynamics simulations were performed using Gromacs 2023Gromacs 2023.2 with the receptor topology using the AMBER99SB-ILDN force field and the ligand topology constructed from GAFF2 force field parameters. Pre-processing such as energy minimization, isothermal isovolumic (NVT) and isothermal isobaric (NPT) equilibrium were performed with a run time of 100 ns and a time step of 2 fs.

## 3 Results

### 3.1 Screening and functional analysis of differential genes

**FIGURE 1:**
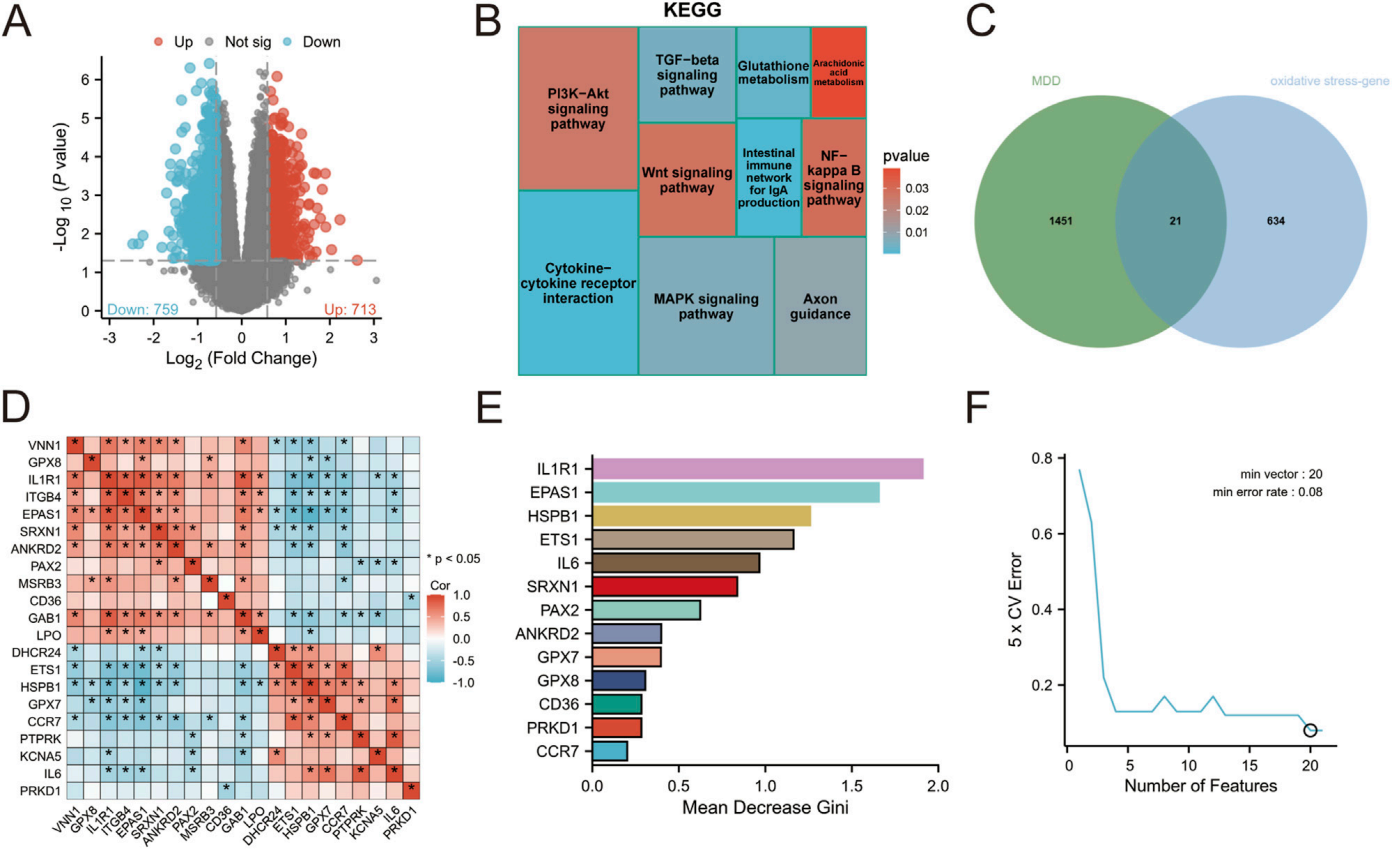
**(A)** Visualization of differential gene volcano map of GSE76826 **(B)** KEGG enrichment analysis of differential genes **(C)** Intersection analysis of depression and oxidative stress **(D)** Network interaction of intersection genes **(E)** Random Forest **(F)** Support Vector Machine.

The analysis identified a total of 1,472 differentially expressed genes, with 713 genes upregulated and 759 genes downregulated (Figure 1A). These differential gene expression patterns were visualized using volcano plot analysis. To gain insights into the potential biological functions associated with these differentially expressed genes, KEGG enrichment analysis was conducted. Notably, the enriched pathways included glutathione metabolism, arachidonic acid metabolism, MAPK signaling pathway, and NF-kappa B signaling pathway. Of particular interest is glutathione (GSH), which plays a crucial role as an antioxidant in cellular defense against reactive oxygen and nitrogen species while maintaining redox homeostasis. Collectively, these findings suggest that the observed differential gene expression may be implicated in OS and inflammation (Figure 1B).

### 3.2 Identification of candidate genes

A total of 655 genes related to OS were screened (Supplementary Table S1), out of which 21 genes were found to be associated with depression (Figure 1C; Supplementary TABLE S2). Correlation analysis was performed on these 21 genes, revealing a significant correlation among them as depicted in Figure 1D. To achieve more accurate classification between the disease group and normal group, two analytical methods, namely, support vector machine (SVM) and random forest were employed for processing. The impact of each gene on the heterogeneity of observations at each node in the classification tree is assessed through random forest analysis. The findings reveal that 13 genes significantly contribute to the classification of depression (Figure 1E; Supplementary Table S3). In support vector machine analysis, a set of 20 feature variables achieved the lowest error rate of 0.08, indicating their importance. Notably, IL1R1 exhibited the lowest score in cross-validation, suggesting its highest significance in classification (Figure 1F; Supplementary Table S4).

### 3.3 Marker screening for patients with depression

**FIGURE 2:**
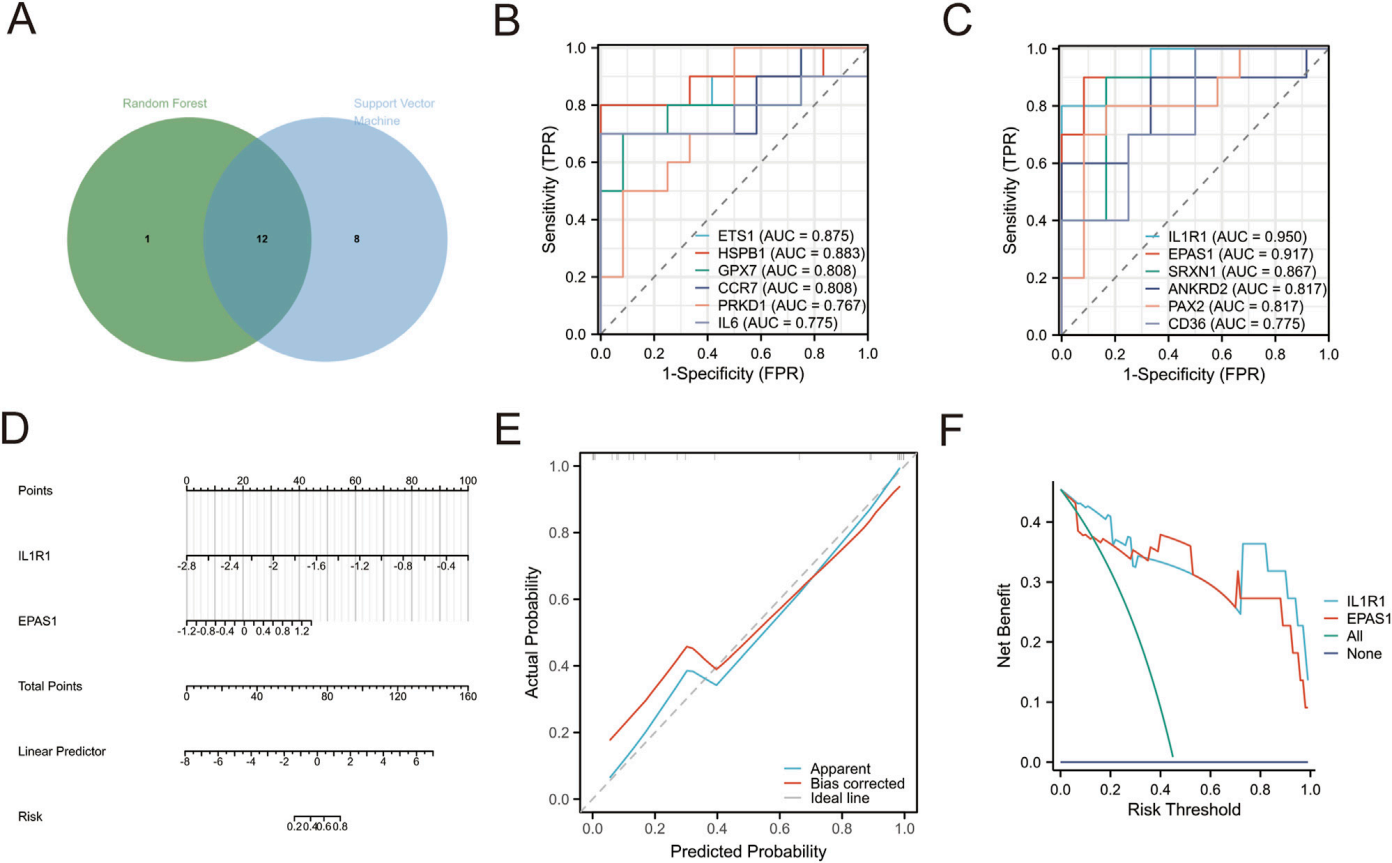
**(A)** Intersection target selected by random forest and support vector machine **(B,C)** ROC diagnostic analysis **(D)** Diagnostic Nomogram. Points: indicates the single score corresponding to each predictive variable under different values. Total Points: indicates the total score of the individual points that correspond to the values of all variables. Linear Predictor: Linear predictor. **(E)** Diagnostic Calibration. Apparent curve indicates prediction curve, Bias-corrected curve indicates calibration curve, and Ideal curve indicates ideal curve **(F)** diagnostic DCA curv

The two machine learning techniques identified a set of twelve genes (IL1R1, EPAS1, HSPB1, ETS1, IL6, SRXN1, PAX2, ANKRD2, GPX7, CD36, PRKD1 and CCR7) that exhibited significant potential for classification (Figure 2A). To further evaluate the diagnostic value of these genes we employed a ROC diagnostic model. Our findings revealed that both ILIRI (AUC = 0.950) and EPASl (AUC = 0.917) demonstrated robust diagnostic power for depression (Figures 2B,C; Supplementary Table S5). Furthermore, we validated this using a Diagnostic Nomogram, where we assessed the probability of a patient being diagnosed with depression by integrating the scores of the IL1R1 and EPAS1 genes (Figure 2D). The calibration curve also confirmed the excellent predictive ability of our ROC model (Figure 2E). Additionally, The DCA visually demonstrates that these tools and models can improve the efficiency of the diagnosis and treatment of depression (Figure 2F). Collectively, the results suggest that both EPASl and ILIRI hold promise as biomarkers for depression with ILIRI exhibiting superior diagnostic capability.

### 3.4 Relationship between key genes and depression

**FIGURE 3:**
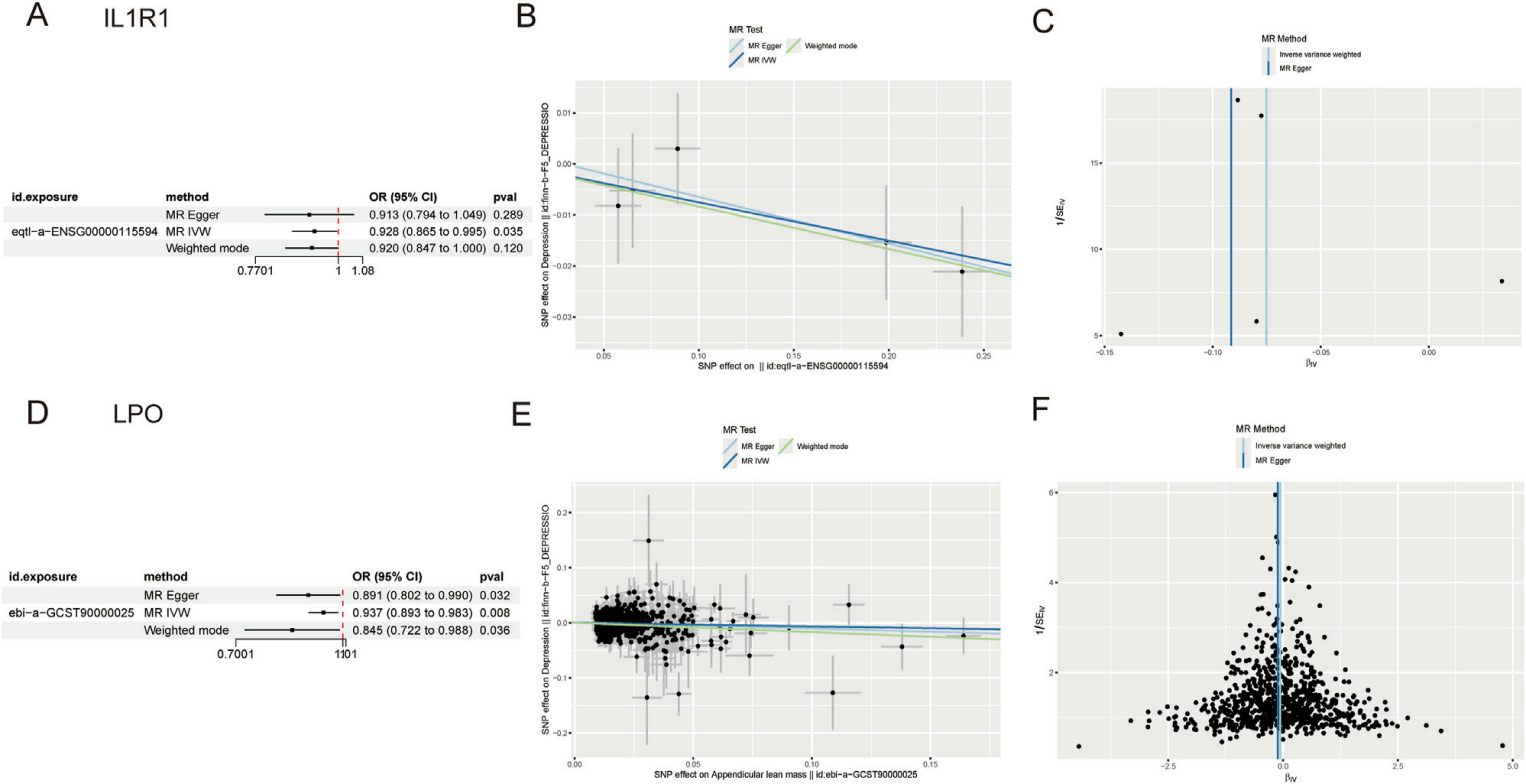
**(A)** Forest plot of IL1R1 **(B)** Scatter plot of IL1R1 **(C)** Funnel plot of IL1R1 **(D)** Forest plot of LPO **(E)** Scatter plot of LPO **(F)** Funnel plot of LPO.

Prospective MR results demonstrated that the key genes IL1R1 (ENSG00000115594, OR = 0.928; 95% CI, 0.865 to 0.995; *P* = 0.035) and LPO (ENSG00000167419, OR = 0.937; 95% CI, 0.893 to 0.983; *P* = 0.008) were significantly associated with a decreased risk of depression, suggesting their potential as protective factors against depression development (Figure 3). However, reverse MR analysis did not reveal any evidence of a causal relationship between depression and these key genes. To assess the stability of our findings, we conducted an evaluation for horizontal pleiotropy using the MR Egger intercept test which yielded a non-significant result (*P* > 0.05), indicating no presence of horizontal pleiotropy bias in our study. Furthermore, through leave-one-out analysis, we found that none of the individual SNPs included in this study had a substantial impact on the robustness of our results, thus confirming their stability (Supplementary Figures S1, S2).

### 3.5 Expression pattern of IL1R1 in brain tissue

**FIGURE 4:**
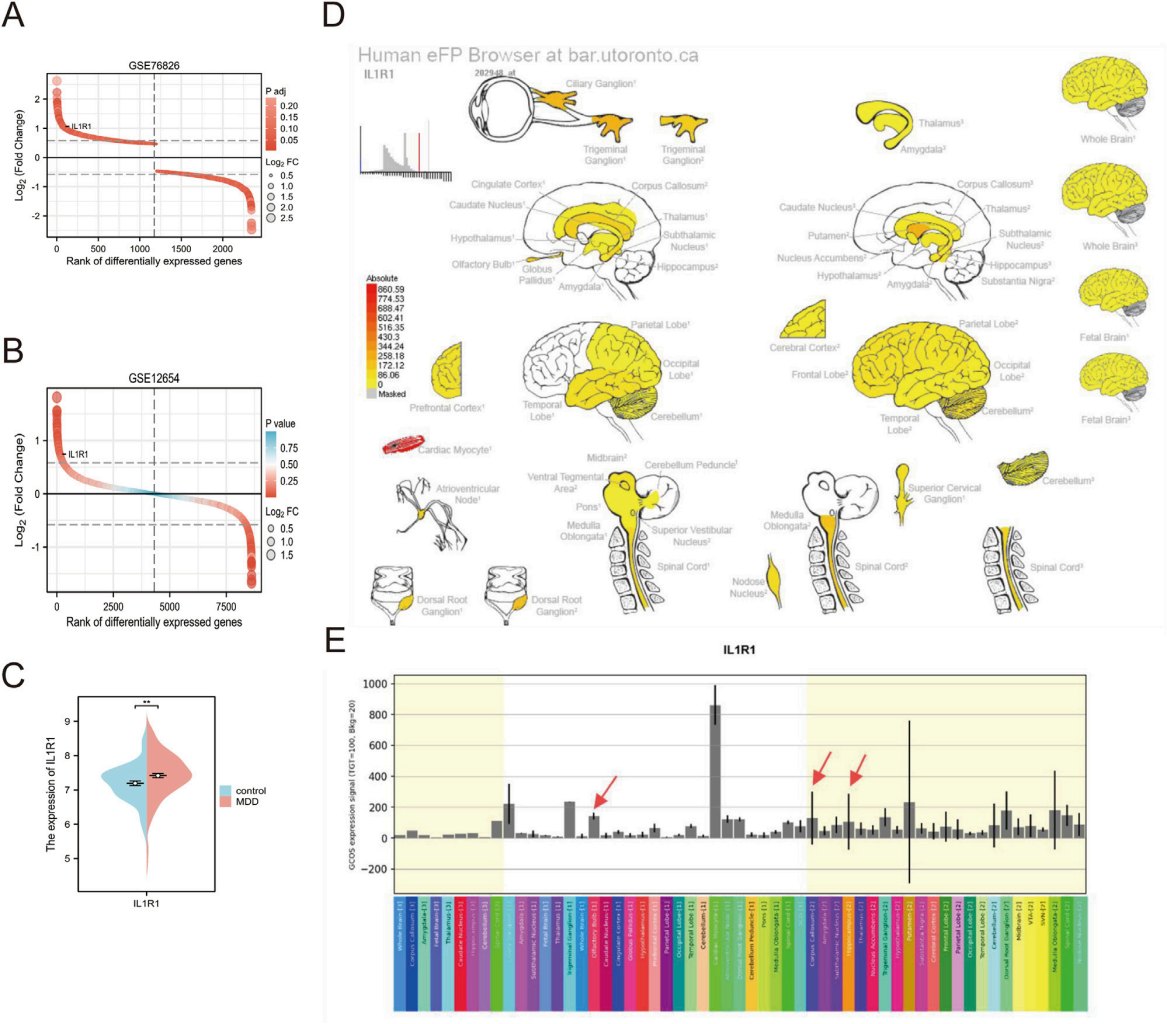
**(A)** Expression of IL1R1 in GSE76826 **(B)** Expression of IL1R1 in GSE12654 **(C)** Expression of IL1R1 in GSE98793 **(D)** Strong expression levels in the olfactory bulb, corpus callosum, and hippocampus of brain tissue are indicated by red arrows **(E)** histogram of expression in brain tissue.

The analysis of the GSE76826 dataset revealed a significant upregulation of IL1R1 expression. (Figure 4A). To further validate this finding, we examined the expression pattern of IL1R1 in the prefrontal cortex using the GSE12654 dataset (Figure 4B). Consistently, our results demonstrated a pronounced elevation of IL1R1 expression in the prefrontal cortex. Similarly, the GSE98793(64 normal individuals and 128 individuals with depression) dataset found a high expression pattern of IL1R1 in peripheral blood (Figure 4C). Moreover, utilizing the Human eFP Browser tool, we investigated the expression profile of IL1R1 in various brain tissues and observed its prominent enrichment in the olfactory bulb, corpus callosum, and hippocampus (Figure 4D). Intriguingly, cardiac muscle cells and trigeminal ganglia exhibited particularly elevated levels of IL1R1 (Figure 4E).

### 3.6 IL1R1 is associated with blood lipids

**FIGURE 5:**
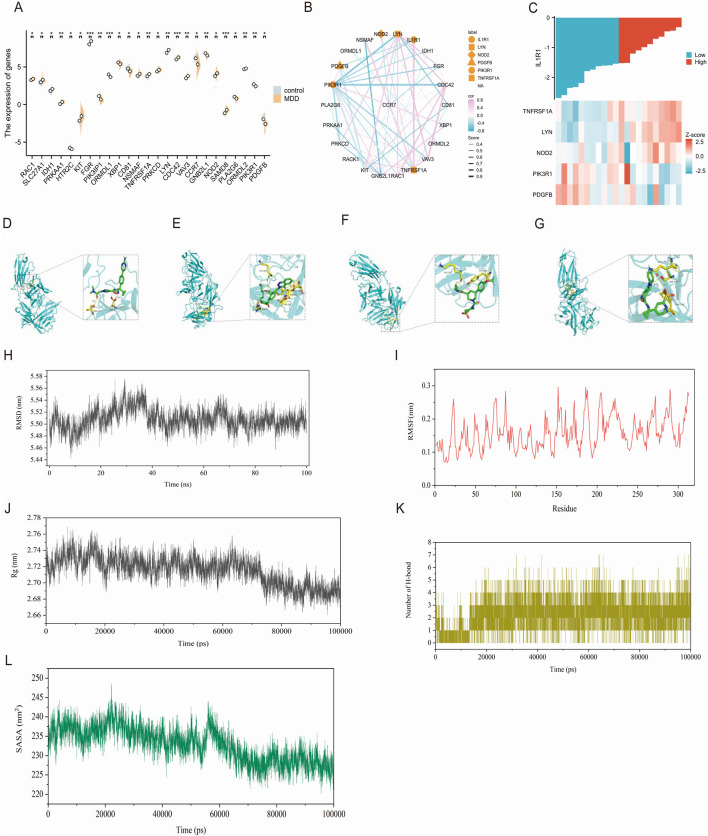
**(A)** The expression of genes related to lipid metabolism **(B)** IL1R1 is correlated with lipid metabolism, pink indicates positive correlation and blue indicates negative correlation. The thickness indicates the score between genes **(C)** indicates the heat map that is correlated with IL1R1 in Figure 1B **(D)** IL1R1 and BI 665915 docking visualization **(E)** IL1R1 and BAY-7598 docking visualization **(F)** IL1R1 and BAY-386 docking visualization **(G)** Visualization of the connection between IL1R1 and BI 639667 **(H)** The atomic root-mean-square deviation (RMSD) of the BI 639667 and IL1R1 complex **(I)** The root-mean-square fluctuation (RMSF) of the BI 639667 and IL1R1 complex **(J)** The radius of gyration (Rg) of the BI 639667 and IL1R1 complex **(K)** The number of hydrogen bonds in the BI 639667 and IL1R1 complex **(L)** The solvent-accessible surface area (SASA) of the BI 639667 and IL1R1 complex.

Dyslipidemia is closely associated with depression, a common condition in patients with dyslipidemia that affects brain lipid metabolism and potentially impacts mood and mental health. In patients with depression, the downregulation of CCR7, GNB2L1, PLA2G6, PIK3IP1, PDGFB, CD81, HTR2C, ORMDL1, and PIK3R1 genes was observed. Conversely, upregulation of NOD2, SAMD, ORMDL2, FGR, KIT, XBP, NSMAF, TNFRSF1, PRKC, LYN, CDC42, VAV3, RAC, SLC27A1, IDH PRKAA genes related to lipid metabolism were detected (Figure 5A). IL-1 serves as a crucial pro-inflammatory cytokine that activates intracellular signaling pathways by binding to IL-IRI receptor and triggers inflammation. Prolonged or excessive inflammatory responses may negatively impact lipid metabolism. Therefore, we identified associations between IL-IRI and TNFRSFIA, NODZ, PDGFB, LVN, and PIK3Rl through the STRING database (Figure 5B). Negative correlations were found between PDGFB and PIK3Rl while positive correlations were observed for TNFRSFIA, NODZ, and LYN (Figure 5C).

### 3.7 IL1R1-related drug screening and molecular docking

The DrugnomeAI analysis revealed a percentile score of 95.87 for IL1R1, indicating its potential medicinal properties. To identify potential drug candidates targeting IL1R1, we conducted a comprehensive gene-drug search. Table 1 presents the small molecule drugs that were selected after rigorous screening and evaluation. These include Epoetin alfa, Anakinra, Tretinoin, Vitamin D, Genistein, BAY-1797, BAY-474, BI 665915, BAY-7598 and BAY-386. In addition to this, Venlafaxine and Paroxetine, which are marketed for the treatment of depression, were selected as positive drugs for affinity comparison. Molecular docking studies were performed using IL1R1 (PDB ID:4gaf) and the aforementioned small molecules to assess their binding energies with IL1R1. The results demonstrated strong interactions between these drug candidates and IL1R1 as evidenced by binding energies below −7; thus suggesting their potential efficacy in treating depression. Whereas, the docking scores with both Venlafaxine and Paroxetine were greater than −7.0, indicating that these drug candidates showed better affinity. Among them, BI 665915, BAY-7598, BAY-386, and BI 639667 exhibited the lowest binding energies and were further visualized through docking simulations with ILIRI (Figures 5D–G). To verify the binding stability, we performed 100 ns molecular dynamics simulations of BI 639667 with IL1R1. The root mean square deviation (RMSD) results showed that the fluctuations of the system were in the range of 0.1 nm, which indicated that the system had strong stability (Figure 5H). The root mean square fluctuation (RMSF) could reflect the overall amino acid residue fluctuation of the complex was small (Figure 5I). The radius of gyration (Rg) suggested that the complexes remained relatively stable during the simulation (Figure 5J). The solvent accessible surface area (SASA) indicated that the binding of BI 639667 had less effect on the structure of IL1R1 (Figure 5K). Further calculation of the hydrogen bonding contacts between BI 639667 and IL1R1 was consistent with the docking results, and the complexes were able to form stable hydrogen bonds (Figure 5L).

**TABLE 1:** Potential drug candidates and docking binding energy of IL1R1.

Database provider	Molecule name	Regulatory approval	Binding energy
Dgidb	Epoetin alfa	Approved	−8.0
	Anakinra	Approved	−7.4
	Tretinoin	Approved	−7.8
	Vitamin D	Approved	−7.0
	Genistein	Approved	−8.0
CHEMBL	BAY-1797	Approved	−8.0
	BAY-474	Approved	−7.4
	BI 665915	Approved	−8.1
	BAY-7598	Approved	−8.4
	BAY-386	Approved	−8.1
	BI 639667	Approved	−9.7
—	Venlafaxine	Approved	−6.1
—	Paroxetine	Approved	−6.7

## 4 Discussion

In the context of depression, there exists a close association between oxidative stress and lipid metabolism. Oxidative stress can induce lipid peroxidation, leading to cellular membrane structural damage and impairment of cell function. Simultaneously, dysregulated lipid levels such as elevated triglyceride levels and increased low-density lipoprotein cholesterol are prone to triggering oxidative stress responses. These two mechanisms interact synergistically, exacerbating the progression of depression. Furthermore, compromised antioxidant defense systems and reduced levels of antioxidant substances render patients more susceptible to oxidative stress-induced damage. Additionally, oxidative stress and lipid abnormalities can facilitate inflammatory responses that further impact the physiological processes underlying depression. Given the intricate etiology of depression and the lack of effective biomarkers for clinical diagnosis, this study employed multiple data analysis methods to screen and validate genes associated with oxidative stress while delving into the mechanistic role played by IL1R1 gene in depth. The findings demonstrate that IL1R1 along with its related genes play a pivotal role in initiating depression onset while also exhibiting potential as emerging biomarkers and therapeutic targets, thereby providing novel insights for scientific research endeavors as well as clinical treatment.

The differentially expressed genes in depression were found to be enriched in metabolic and inflammatory pathways, with a notable implication of glutathione metabolism suggesting their association with oxidative stress. These findings are consistent with previous studies (Liu et al., 2020; Savushkina et al., 2022) and further support the notion that metabolic disorders serve as important biomarkers for depression. Subsequent investigations revealed a total of 12 genes linked to OS, among which EPAS and IL1R1 were selected as potential diagnostic biomarkers. Endothelial PAS1 protein (EPAS1), also known as hypoxia-inducible factor 2α (HIF-2α), is involved in regulating the transition from acute to chronic hypoxia and subsequently influences the expression of inducible nitric oxide synthase (iNOS). Elevated levels of iNOS have been implicated in MDD. In addition, Qing Wu et al. reported that HIF-2α can suppress adipose tissue thermogenesis through modulation of intestinal microbiota (Wu et al., 2021). Interleukin-1 receptor type 1 (IL1R1) is a crucial component of the immune response, mediating the effects of interleukin-1 (IL-1) in the inflammatory process. This study observed an increased expression of IL1R1 in both peripheral blood and brain tissues of patients with depression, suggesting that IL1R1-mediated signaling may play a role in neuroinflammatory states associated with this condition (Liu et al., 2019; Song et al., 2023). Research conducted by Ja Wook Koo and Carol L. Murray et al. demonstrated that mice lacking IL-1R1 exhibited anti-anxiety behaviors (Koo and Duman, 2009; Murray et al., 2013). Subsequently, we employed Mendelian randomization analysis to identify IL1R1 and LPO as protective factors against depression. Existing literature indicates that neuronal IL-1R1 regulates gene pathways involved in synaptic organization without eliciting typical inflammatory responses (Nemeth et al., 2024). Furthermore, genetic studies have identified polymorphisms within the IL1R1 gene associated with responses to antidepressant therapy, implying that IL1R1 may influence the efficacy of therapeutic interventions (Tsai et al., 2023). These findings underscore the dual role of ILIR2 in depression and its potential clinical significance. Given our results indicating limited diagnostic capacity for LPO in depression, subsequent analysis focused on ILIR. In this study, we utilized the Human eFP Browser online tool to analyze the expression patterns of the IL1R1 gene across various tissues, with a specific focus on its expression within the brain. Our study findings suggest that although IL1R1 exhibits the highest expression level in cardiomyocytes and trigeminal ganglia, our targeted analysis of specific brain regions reveals that localized overexpression of IL1R1 in the olfactory bulb, corpus callosum, and key brain regions (Ashmun et al., 1986) indicates its potential role in regulating neurobiological processes to counteract depression development. Previous research has demonstrated the crucial involvement of IL-1β/IL-1R1 signaling in the olfactory bulb for inducing and propagating abnormal α-Syn (Niu et al., 2020).

In the aforementioned study, it was discovered that upregulated genes associated with depression are implicated in metabolism, and OS interacts with lipid metabolism. Consequently, our analysis uncovers a connection between IL1R1 and lipid regulation, offering valuable insights into the metabolic facets of depression. Utilizing the STRING database, we observed a negative correlation between IL1R and PDGFB as well as PIK3R1, while a positive correlation was found with TNFRSF1A, NOD2, and LYN. The differential expression of several pivotal genes underscores dysregulation of lipid metabolism in depression. Notably among them is PDGFB (platelet-derived growth factor subunit B), which plays a crucial role in various cellular processes such as cell proliferation, survival, and migration—particularly within the realm of vascular biology and neurodevelopment (Bi et al., 2022). The upregulation of PIK3R1 in patients with depression may indicate a compensatory mechanism for lipid profile changes that could impact the neuroinflammatory pathway mediated by PIK3R1, a regulatory subunit 1 of inosine-3 kinase (PI3K) signaling pathway. This pathway is crucial for various cellular functions, including metabolism, growth, and survival. The downregulation of PIK3R1 in depressed patients may disrupt these processes, leading to impaired lipid metabolism and exacerbation of depressive symptoms. Previous studies have demonstrated the involvement of PI3K signaling in mood regulation and emotional responses, suggesting that alterations in this pathway might contribute to the pathophysiology of depression (Guo et al., 2024). Additionally, TNFRSF1A (Tumor necrosis factor receptor superfamily member 1A), as a receptor for tumor necrosis factor α (TNF-α), deserves attention due to its significant role in mediating inflammatory responses. In the context of depression, its upregulation may indicate an enhanced inflammatory state associated with various neuropsychiatric disorders. Our STRING database analysis revealed an interaction between TNFRSF1A and IL1R1 which suggests the existence of a complex inflammatory signaling network that could further influence lipid metabolism. Overall, these findings emphasize the intricate relationship between lipid metabolism and inflammatory pathways within the context of depression and warrant further investigation into their therapeutic implications. Although oxidative stress and lipid metabolism are significant contributors to the pathophysiology of depression, they represent only two among numerous biological pathways that may be implicated. Future research must adopt a multidisciplinary, multifaceted systems biology approach to comprehensively elucidate the underlying causes of depression.

DrugnomeAI identified IL1R1 as a potential drug target. Molecular docking results revealed strong binding affinity (with binding energy lower than −7 kcal/mol) between IL1R1 and several small molecules, including BI 665915, BAY-7598, BAY-386, and BI 639667, suggesting their potential therapeutic effects. The molecular dynamics simulation results of 100ns showed strong binding stability between IL1R1 and BI 639667. Epoetin Alfa is a recombinant erythropoietin (EPO) traditionally used for anemia treatment; however, it has been discovered that both EPO and its receptor (EPO-R) are widely expressed in the central and peripheral nervous systems. Moreover, they can traverse the blood-brain barrier to exert nutritional and protective effects within the central nervous system (Miskowiak et al., 2012). Kamilla W Miskowiakt et al. investigated the impact of combining electroconvulsive therapy with erythropoietin (EPO) on enhancing cognitive performance in patients with depression and mood disorders (Miskowiak et al., 2024). Additionally, Anakinra, a recombinant IL-1 receptor antagonist, has demonstrated therapeutic potential in various inflammatory conditions and may offer a novel treatment approach for depression by mitigating the effects of pro-inflammatory cytokines (Pazyar et al., 2012). However, it is important to acknowledge certain limitations within this study. The analysis solely relied on bioinformatics methods without incorporating data from wet laboratory experiments to provide more direct evidence supporting the underlying biological mechanisms involved. Currently, there are no available investigation results regarding the effects of BAY-386 and BI 639667 compounds on depression.

In summary, this study successfully identified key genes associated with depression through comprehensive bioinformatics analysis, encompassing differential gene expression, GO and KEGG enrichment analyses, and network interaction analysis. Furthermore, the validity of these genes as biomarkers for depression was assessed using a Diagnostic Nomogram, while MR was employed to explore the causal relationship between depression and these key genes. Additionally, potential drug targets were identified, offering novel avenues for therapeutic intervention. These findings provide valuable insights into the molecular mechanisms underlying depression and pave the way for future research and clinical applications. By addressing limitations in subsequent studies, these results could significantly contribute to the development of more effective treatments for depression.

## Data Availability

The original contributions presented in the study are included in the article/Supplementary Material, further inquiries can be directed to the corresponding authors.

## Supplementary material

SUPPLEMENTARY TABLE S1 Oxidative stress-related genes.

SUPPLEMENTARY TABLE S2 Correlation of intersecting genes.

SUPPLEMENTARY TABLE S3 Random forest- importance of feature variables.

SUPPLEMENTARY TABLE S4 Support vector machine- feature score sheet.

SUPPLEMENTARY TABLE S5 Statistical description of ROC curve.

SUPPLEMENTARY FIGURE S1 The leaveOneOut plot of IL1R1.

SUPPLEMENTARY FIGURE S2 The leaveOneOut plot of LPO.

## References

[B1] Ait Tayeb A. E. K. Poinsignon V. Chappell K. Bouligand J. Becquemont L. Verstuyft C. (2023). Major depressive disorder and oxidative stress: a review of peripheral and genetic biomarkers according to clinical characteristics and disease stages. Antioxidants (Basel) 12, 942. 10.3390/antiox12040942 37107318 PMC10135827

[B2] Ashmun R. A. Hultquist D. E. Schultz J. S. (1986). Kinetic analysis in single, intact cells by microspectrophotometry: evidence for two populations of erythrocytes in an individual heterozygous for glucose-6-phosphate dehydrogenase deficiency. Am. J. Hematol. 23, 311–316. 10.1002/ajh.2830230402 3788959

[B3] Bi Q. Wang C. Cheng G. Chen N. Wei B. Liu X. (2022). Microglia-derived PDGFB promotes neuronal potassium currents to suppress basal sympathetic tonicity and limit hypertension. Immunity 55, 1466–1482.e9. 10.1016/j.immuni.2022.06.018 35863346

[B4] Breiman L. (2001). Random forests. Mach. Learn. 45, 5–32. 10.1023/a:1010933404324

[B5] Brock J. Basu N. Schlachetzki J. C. M. Schett G. Mcinnes I. B. Cavanagh J. (2023). Immune mechanisms of depression in rheumatoid arthritis. Nat. Rev. Rheumatol. 19, 790–804. 10.1038/s41584-023-01037-w 37923863

[B6] Chai Y. Sheline Y. I. Oathes D. J. Balderston N. L. Rao H. Yu M. (2023). Functional connectomics in depression: insights into therapies. Trends Cogn. Sci. 27, 814–832. 10.1016/j.tics.2023.05.006 37286432 PMC10476530

[B7] Chen Z. Huang X. Gong Q. Biswal B. B. (2021). Translational application of neuroimaging in major depressive disorder: a review of psychoradiological studies. Front. Med. 15, 528–540. 10.1007/s11684-020-0798-1 33511554

[B8] Cobley J. N. Fiorello M. L. Bailey D. M. (2018). 13 reasons why the brain is susceptible to oxidative stress. Redox Biol. 15, 490–503. 10.1016/j.redox.2018.01.008 29413961 PMC5881419

[B9] Cui L. Li S. Wang S. Wu X. Liu Y. Yu W. (2024). Major depressive disorder: hypothesis, mechanism, prevention and treatment. Signal Transduct. Target Ther. 9, 30. 10.1038/s41392-024-01738-y 38331979 PMC10853571

[B10] Dang R. Wang M. Li X. Wang H. Liu L. Wu Q. (2022). Edaravone ameliorates depressive and anxiety-like behaviors via Sirt1/Nrf2/HO-1/Gpx4 pathway. J. Neuroinflammation 19, 41. 10.1186/s12974-022-02400-6 35130906 PMC8822843

[B11] Fries G. R. Saldana V. A. Finnstein J. Rein T. (2023). Molecular pathways of major depressive disorder converge on the synapse. Mol. Psychiatry 28, 284–297. 10.1038/s41380-022-01806-1 36203007 PMC9540059

[B12] Guo N. Wang X. Xu M. Bai J. Yu H. Le Z. (2024). PI3K/AKT signaling pathway: molecular mechanisms and therapeutic potential in depression. Pharmacol. Res. 206, 107300. 10.1016/j.phrs.2024.107300 38992850

[B13] He L. He T. Farrar S. Ji L. Liu T. Ma X. (2017). Antioxidants maintain cellular redox homeostasis by elimination of reactive oxygen species. Cell Physiol. Biochem. 44, 532–553. 10.1159/000485089 29145191

[B14] Kobayashi N. Shinagawa S. Nagata T. Shigeta M. Kondo K. (2022). Suppressors of cytokine signaling are decreased in major depressive disorder patients. J. Pers. Med. 12, 1040. 10.3390/jpm12071040 35887537 PMC9315526

[B15] Koo J. W. Duman R. S. (2009). Interleukin-1 receptor null mutant mice show decreased anxiety-like behavior and enhanced fear memory. Neurosci. Lett. 456, 39–43. 10.1016/j.neulet.2009.03.068 19429130 PMC3678367

[B16] Liu X. Liu X. Wang Y. Zeng B. Zhu B. Dai F. (2023). Association between depression and oxidative balance score: national health and nutrition examination survey (NHANES) 2005-2018. J. Affect Disord. 337, 57–65. 10.1016/j.jad.2023.05.071 37244542

[B17] Liu X. Nemeth D. P. Mckim D. B. Zhu L. Disabato D. J. Berdysz O. (2019). Cell-type-specific interleukin 1 receptor 1 signaling in the brain regulates distinct neuroimmune activities. Immunity 50, 764–766. 10.1016/j.immuni.2019.02.012 30893590

[B18] Liu Z. Wang J. Liu L. Yuan H. Bu Y. Feng J. (2020). Chronic ethanol consumption and HBV induce abnormal lipid metabolism through HBx/SWELL1/arachidonic acid signaling and activate Tregs in HBV-Tg mice. Theranostics 10, 9249–9267. 10.7150/thno.46005 32802190 PMC7415795

[B19] Lu Z. Pu C. Zhang Y. Sun Y. Liao Y. Kang Z. (2022). Oxidative stress and psychiatric disorders: evidence from the bidirectional mendelian randomization study. Antioxidants (Basel) 11, 1386. 10.3390/antiox11071386 35883877 PMC9312055

[B20] Marwaha S. Palmer E. Suppes T. Cons E. Young A. H. Upthegrove R. (2023). Novel and emerging treatments for major depression. Lancet 401, 141–153. 10.1016/S0140-6736(22)02080-3 36535295

[B21] Meyer D. Dimitriadou E. Hornik K. Weingessel A. Leisch F. (2015). Misc functions of the department of statistics. ProbabilityTheory Group.

[B22] Miskowiak K. W. Petersen J. Z. Macoveanu J. Ysbæk-Nielsen A. T. Lindegaard I. A. Cramer K. (2024). Effect of erythropoietin on cognitive side-effects of electroconvulsive therapy in depression: a randomized, double-blind, placebo-controlled trial. Eur. Neuropsychopharmacol. 79, 38–48. 10.1016/j.euroneuro.2023.12.004 38128460

[B23] Miskowiak K. W. Vinberg M. Harmer C. J. Ehrenreich H. Kessing L. V. (2012). Erythropoietin: a candidate treatment for mood symptoms and memory dysfunction in depression. Psychopharmacol. Berl. 219, 687–698. 10.1007/s00213-011-2511-1 21947319

[B24] Miyata S. Kurachi M. Okano Y. Sakurai N. Kobayashi A. Harada K. (2016). Blood transcriptomic markers in patients with late-onset major depressive disorder. PLoS One 11, e0150262. 10.1371/journal.pone.0150262 26926397 PMC4771207

[B25] Murray C. L. Obiang P. Bannerman D. Cunningham C. (2013). Endogenous IL-1 in cognitive function and anxiety: a study in IL-1RI-/- mice. PLoS One 8, e78385. 10.1371/journal.pone.0078385 24205219 PMC3813582

[B26] Nemeth D. P. Liu X. Monet M. C. Niu H. Maxey G. Schrier M. S. (2024). Localization of brain neuronal IL-1R1 reveals specific neural circuitries responsive to immune signaling. J. Neuroinflammation 21, 303. 10.1186/s12974-024-03287-1 39563437 PMC11575132

[B27] Niu H. Wang Q. Zhao W. Liu J. Wang D. Muhammad B. (2020). IL-1β/IL-1R1 signaling induced by intranasal lipopolysaccharide infusion regulates alpha-Synuclein pathology in the olfactory bulb, substantia nigra and striatum. Brain Pathol. 30, 1102–1118. 10.1111/bpa.12886 32678959 PMC7754320

[B28] Patel M. (2016). Targeting oxidative stress in central nervous system disorders. Trends Pharmacol. Sci. 37, 768–778. 10.1016/j.tips.2016.06.007 27491897 PMC5333771

[B29] Pazyar N. Feily A. Yaghoobi R. (2012). An overview of interleukin-1 receptor antagonist, anakinra, in the treatment of cutaneous diseases. Curr. Clin. Pharmacol. 7, 271–275. 10.2174/157488412803305821 22794157

[B30] Savushkina O. K. Boksha I. S. Omel'chenko M. A. Tereshkina E. B. Prokhorova T. A. Vorobieva E. A. (2022). Activity of enzymes of glutamate, energy and glutathione metabolism in the first juvenile depression with attenuated symptoms of schizophrenia. Zh Nevrol. Psikhiatr Im. S S Korsakova 122, 136–144. 36036415 10.17116/jnevro2022122081136

[B31] Song J. Ma Z. Zhang H. Liang T. Zhang J. (2023). Identification of novel biomarkers linking depressive disorder and Alzheimer's disease based on an integrative bioinformatics analysis. BMC Genom Data 24, 22. 10.1186/s12863-023-01120-x 37061663 PMC10105463

[B32] Sun N. Qin Y. J. Xu C. Xia T. Du Z. W. Zheng L. P. (2022). Design of fast-onset antidepressant by dissociating SERT from nNOS in the DRN. Science 378, 390–398. 10.1126/science.abo3566 36302033

[B33] Syed S. A. Beurel E. Loewenstein D. A. Lowell J. A. Craighead W. E. Dunlop B. W. (2018). Defective inflammatory pathways in never-treated depressed patients are associated with poor treatment response. Neuron 99, 914–924.e3. 10.1016/j.neuron.2018.08.001 30146307 PMC6151182

[B34] Tauffenberger A. Magistretti P. J. (2021). Reactive oxygen species: beyond their reactive behavior. Neurochem. Res. 46, 77–87. 10.1007/s11064-020-03208-7 33439432 PMC7829243

[B35] Tsai S. J. Kao C. F. Su T. P. Li C. T. Lin W. C. Hong C. J. (2023). Cytokine- and vascular endothelial growth factor-related gene-based genome-wide association study of low-dose ketamine infusion in patients with treatment-resistant depression. CNS Drugs 37, 243–253. 10.1007/s40263-023-00989-7 36763263

[B36] Wu Q. Liang X. Wang K. Lin J. Wang X. Wang P. (2021). Intestinal hypoxia-inducible factor 2α regulates lactate levels to shape the gut microbiome and alter thermogenesis. Cell Metab. 33, 1988–2003.e7. 10.1016/j.cmet.2021.07.007 34329568

